# MS-Based Allotype-Specific Analysis of Polyclonal IgG-Fc *N*-Glycosylation

**DOI:** 10.3389/fimmu.2020.02049

**Published:** 2020-08-21

**Authors:** Thomas Sénard, Andrea F. G. Gargano, David Falck, Steven W. de Taeye, Theo Rispens, Gestur Vidarsson, Manfred Wuhrer, Govert W. Somsen, Elena Domínguez-Vega

**Affiliations:** ^1^Center for Proteomics and Metabolomics, Leiden University Medical Center, Leiden, Netherlands; ^2^Van ’t Hoff Institute for Molecular Sciences, University of Amsterdam Analytical Chemistry Group, Amsterdam, Netherlands; ^3^Amsterdam Institute for Molecular and Life Sciences (AIMMS), Vrije Universiteit Amsterdam, Amsterdam, Netherlands; ^4^Department of Experimental Immunohematology, Sanquin Research, Amsterdam, Netherlands; ^5^Department of Immunopathology, Sanquin Research, Amsterdam, Netherlands

**Keywords:** immunoglobulin G, allotypes, fragment crystallizable, *N*-glycosylation, post-translational modifications, mass spectrometry, hydrophilic interaction liquid chromatography, capillary electrophoresis

## Abstract

Current approaches to study glycosylation of polyclonal human immunoglobulins G (IgG) usually imply protein digestion or glycan release. While these approaches allow in-depth characterization, they also result in a loss of valuable information regarding certain subclasses, allotypes and co-occuring post-translational modifications (PTMs). Unfortunately, the high variability of polyclonal IgGs makes their intact mass spectrometry (MS) analysis extremely challenging. We propose here a middle-up strategy for the analysis of the intact fragment crystallizable (Fc) region of human plasma IgGs, with the aim of acquiring integrated information of the *N*-glycosylation and other PTMs of subclasses and allotypes. Human plasma IgG was isolated using Fc-specific beads followed by an on-bead C_*H*_2 domain digestion with the enzyme IdeS. The obtained mixture of Fc subunits was analyzed by capillary electrophoresis (CE) and hydrophilic interaction liquid chromatography (HILIC) hyphenated with MS. CE-MS provided separation of different IgG-subclasses and allotypes, while HILIC-MS allowed resolution of the different glycoforms and their oxidized variants. The orthogonality of these techniques was key to reliably assign Fc allotypes. Five individual donors were analyzed using this approach. Heterozygosis was observed in all the analyzed donors resulting in a total of 12 allotypes identified. The assignments were further confirmed using recombinant monoclonal IgG allotypes as standards. While the glycosylation patterns were similar within allotypes of the same subclass, clear differences were observed between IgG subclasses and donors, highlighting the relevance of the proposed approach. In a single analysis, glycosylation levels specific for each allotype, relative abundances of subclasses and information on co-occurring modifications are obtained. This middle-up method represents an important step toward a comprehensive analysis of immunoglobulin G-Fc variants.

## Introduction

Immunoglobulins (Ig) are glycoproteins produced by plasma cells which play a key role in the adaptive immune system. Immunoglobulin G (IgG) represents the most abundant class of Ig in human plasma and consists of a variable domain [F(ab)_2_] and a fragment crystallizable (Fc)-region. Four subclasses of IgGs (1–4) can be distinguished based on the Fc region and differ in structure, abundance, and effector functions (1). Subclass-specific analysis of IgGs is highly relevant for understanding the role of IgGs in diseases, for biomarkers discovery, and to design therapeutic interventions. This is especially true for low abundant subclasses. For instance, IgG4 antibodies are associated with specific diseases (2, 3), and IgG3 has potential for the development of therapeutics and vaccines (4). For each of the subclasses a range of allotypes have been described, which show a slight variation in the amino acid sequences of the constant region of heavy or light chains between individuals (5). Importantly, IgG allotypes were associated with various cancers (6–8) and changes in the immune response (9, 10). Recently, different allotypes, especially of IgG3, have been found to differ in affinities for Fcγ receptors (11), key mediators of IgG effector function (12).

The Fc region possesses an *N*-glycosylation site on Asn297 of each heavy chain, occupied with a diantennary complex type glycan. These glycans are highly heterogeneous with compositions differing in the presence or absence of a core fucose or bisecting *N*-acetylglucosamine (GlcNAc) as well as the number of galactose and *N*-acetylneuraminic acid residues in the *N*-glycan antennae. Many studies of plasma IgG *N*-glycosylation have demonstrated the relevance of Fc glycosylation for the immune system, notably by its involvement in the binding to the Fcγ receptors (13, 14). For instance, in the case of the Fcγ receptor IIIa, the lack of core fucose on IgG glycans increases the binding affinity up to 100-fold (15). The pattern of IgG Fc glycosylation depends on different factors, such as genetics, epigenetics and aging but also has been associated with various pathologies including inflammation and cancer (16, 17).

In addition to glycosylation, IgGs may exhibit many other post-translational modifications (PTMs) in the Fc region which can also influence effector functions. The most frequent ones are C-terminal lysine-clipping required for the activation of the complement (18) and oxidation, which impairs the binding to the neonatal Fc receptor (FcRn) implicated in the recycling of IgGs (19). Other PTMs can occur during pathological conditions. For instance, carbamylation and citrullination have been observed in patients with rheumatoid arthritis and can alter complement activation (20) and binding to Fcγ receptors (21).

The high heterogeneity of IgGs comprising subclasses, allotypes and PTMs, raises the question as to their potential interplay. As an example, specific glycosylation profiles can be associated with specific IgG amino acid sequences (22) including subclasses (23) and certain allotypes (11). In-depth characterization of this heterogeneity is therefore much needed but also challenging. A common and powerful analytical technique to study proteins and their PTMs is mass spectrometry (MS) (24). The MS analysis of monoclonal antibodies (mAbs) can be performed at the intact level providing information on glycosylation and other co-occurring modifications (25, 26). However, due to the complexity and inherent variability of polyclonal IgGs with over one million different antibody species in plasma (based on amino acid sequences only), their intact analysis is extremely challenging. Current approaches to study polyclonal human IgGs, therefore, include protein digestion (bottom-up approaches) or, in the particular case of glycosylation, glycan release followed by derivatization or labeling (27–29). Although these methods provide detailed information on glycosylation, their use inevitably brings a substantial loss of information toward subclasses, sequence variants (i.e., allotypes) and co-occurring PTMs. For instance, glycopeptides obtained for IgG2 and IgG3 share the same amino acid sequence (30) as is true for many allotypes (1). Goetze et al. (31) investigated the possibility of screening polyclonal IgG-Fc modifications through reversed phase liquid chromatography (RPLC)-MS of individual Fc heavy chains (Fc/2 subunits). After several purifications, digestions and analysis, they resolved IgG1 and IgG2 allotypes with modifications, such as glycosylation with a focus on fucosylation and glycation. Nevertheless, they did not achieve the resolution of IgG3 and IgG4 allotypes and, therefore, also not a comprehensive characterization of polyclonal IgGs in a single analysis.

It is known that the Fc region carries most of the information regarding the isotypes and allotypes in their C_*H*_2 and C_*H*_3 domains (1, 5) and exhibit various modifications that define the structure and functions of IgGs (32). Therefore, MS analysis of individual Fc subunits can serve as an alternative to intact IgG characterization in polyclonal samples, reducing the complexity of the variable domain but providing integrated information on key structural features. As Fc subunits still comprise a diverse mixture of subclasses, allotypes and PTMs with very similar masses, they cannot be resolved with MS only. Separation of these fragments prior to MS detection can reduce complexity and add confidence to the assignments. Among the separation techniques which are MS compatible, hydrophilic interaction liquid chromatography (HILIC) and capillary electrophoresis (CE) are powerful tools for the analysis of intact proteins (33, 34). CE provides a charge-based separation while in HILIC, the selectivity is dominated by analyte polarity providing separation of PTMs such as glycosylation and oxidation. For these reasons, these techniques are often employed for the profiling of intact glycoproteins (35), including therapeutic antibodies (36–38).

Here, we present a new middle-up strategy for MS analysis of intact Fc subunits from human plasma IgGs, with the aim of acquiring a comprehensive overview of the glycosylation and other PTMs of each specific allotype. The antibodies were captured from plasma of single donors using C_*H*_3 domain-specific beads. The IgGs were then digested on-bead using the IdeS enzyme. This allowed isolation of the Fc/2 subunits and subsequent MS analysis of the intact subdomains. Prior to MS analysis, two orthogonal separation techniques, HILIC and CE, have been explored for the separation of the isolated Fc/2 subunits. The proposed approach permits a comprehensive analysis of Fc proteoforms and allows to study the glycosylation levels specific for each subclass and allotype as well as their relationships.

## Materials and Methods

### Materials

IdeS enzyme (FabRICATOR^®^) of *Streptococcus pyogenes* was purchased from Genovis AB (Lund, Sweden). Sequencing grade modified trypsin was obtained from Promega (Madison, WI, United States). Milli-Q deionized water (MQ) was generated using a Q-Gard 2 system (Millipore, Amsterdam, Netherlands) maintained at ≥18.2 MΩ. MS grade acetonitrile (ACN) and trifluoroacetic acid (TFA) were acquired from Biosolve BV (Valkenswaard, Netherlands). Analytical grade formic acid (FA), acetic acid (AA), trizma hydrochloride, Tris(hydroxymethyl)aminomethane, sodium deoxycholate (SDC), 2-chloroacetamide (CAA) and tris(2-carboxyethyl)phosphine (TCEP) were obtained from Sigma-Aldrich (Steinheim, Germany). LC-MS grade water was purchased from Fluka (Steinheim, Germany). Ammonium bicarbonate (ABC), LC-MS grade methanol (MeOH), disodium hydrogen phosphate dihydrate (Na_2_HPO_4_, 2 H_2_O), potassium dihydrogen phosphate (KH_2_PO_4_), and sodium chloride (NaCl) were obtained from Merck (Darmstadt, Germany). Phosphate-buffered saline (PBS, 0.035 M, pH 7.6) was prepared in-house with 5.7 g/L of Na_2_HPO_4_, 2 H_2_O, 0.5 g/L of KH_2_PO_4_, and 8.5 g/L of NaCl.

### Samples

Plasma samples were collected from five different healthy donors in accordance with Dutch regulations and after approval from the Sanquin Ethical Advisory Board in accordance with the Declaration of Helsinki.

Production and characterization of IgG allotypes was previously described by de Taeye et al. (11) and the exact same batch of monoclonal anti-RhD IgG allotypes was used for our MS analysis. In short, monoclonal standard IgG allotypes with anti-RhD specificity were produced in HEK293F cells following the protocol described by Dekkers et al. (13). Antibodies were then purified using a HiTrap protein A or protein G column and eluted with phosphate citrate buffer pH 3.0 for IgG1, IgG2, and IgG4 allotypes and 0.1 M glycine pH 2.5–3.0 for IgG3 allotypes. After dialysis, they were stored in Nanogam buffer (5% D-glucose, 5 mM Sodium acetate, pH 4.5) at −20°C.

### IgG Purification and Digestion

IgGs were captured from plasma following the protocol described by Bondt et al. (39). Briefly, 50 μg of IgGs from plasma (5 μL to 20 μL) or 25 μg of each monoclonal standard IgG allotype (9.5 μL to 104.17 μL), were captured using 10 μL or 5 μL of anti-human Fc agarose beads (CaptureSelect^TM^ FcXL Affinity Matrix, Thermo Fisher Scientific, Naarden, Netherlands), respectively, on a 96-well filter plate (10 μm pore, Orochem Technologies, Naperville, IL). Prior to incubation with the samples, the beads were washed thrice with 200 μL of PBS on a vacuum manifold. Afterward, the samples, diluted in 100 μL of PBS, were added to the beads and incubated for 1 h at room temperature on a multi-well shaker plate (VWR, Amsterdam, Netherlands) at 1000 rpm. The remaining plasma was then removed by centrifugation at 50 x *g* for 1 min, and the beads were washed four times with 200 μL of PBS.

After capturing, IgGs were digested on the beads using the recombinant streptococcal IdeS enzyme following the protocol described by the manufacturer. For that purpose 1 U of enzyme was added per μg of sample in a total volume of 35 μL of PBS. After addition of the enzyme, the beads were shaken for 5 min at room temperature and incubated overnight at 37°C, in a humid environment to prevent drying. After incubation, the Fab subunits were removed by centrifugation at 50 x *g* for 1 min. The Fc subunits, still on the beads, were then washed thrice with 200 μL of PBS and 200 μL of MQ. To remove the Fc subunits from the beads, 100 μL of 100 mM of FA were added. The filter plate was then placed on a shaker plate at 1000 rpm and at room temperature for 5 min. Finally, Fc subunits were eluted by centrifugation at 50 x *g* for 1 min into V-bottom plates (Greiner Bio-One, Frickenhausen, Germany). After equal division of the samples into two V-bottom plates (for CE-MS and HILIC-MS analysis), the Fc subunits were dried by vacuum centrifugation in a rotational vacuum concentrator (RVC 2-25 CDplus, Christ, Osterode, Germany) set up at 50°C for 2.5 h.

### SDS-PAGE

The sample purity, the beads binding capacity and the enzyme efficacy were assessed by SDS-PAGE. Shortly, 5 μg of Fc subunits were dissolved in a non-reducing loading buffer (NuPAGE^TM^ LDS Sample buffer, Thermo Fisher Scientific) and denaturated at 60°C for 5 min. They were then applied on the wells of the gel with protein standards (Precision Plus Protein^TM^ All Blue Prestained Protein Standards, Bio-Rad, Veenendaal, Netherlands). Separation was performed on a 4–12% Bis-Tris gel (NuPAGE^TM^ Novex^TM^, Thermo Fisher Scientific) in a MOPS SDS running buffer (NuPAGE^TM^, Thermo Fisher Scientific), at 200 V constant voltage for 55 min. After Coomassie blue staining (SimplyBlue^TM^ SafeStain, Thermo Fisher Scientific), the gel was visualized under trans-illumination using an Amersham Imager 600 (GE Healthcare).

### HILIC-MS Analysis

Prior HILIC experiments, IgGs from human plasma or monoclonal antibodies were dissolved in 100 mM FA in water to a concentration of 1 mg/mL, and then diluted with MQ water to 0.083 mg/mL or 0.042 mg/mL, respectively.

HILIC separations were performed on an UltiMate RSLCnano system (Thermo Fisher Scientific, Breda, Netherlands), which comprises an autosampler with a 20 μL loop and a thermostated column compartment at 50°C. The capillary amideHILIC column was packed using the stationary phase obtained from unpacking a HILIC column (Agilent AdvanceBio glycan mapping column, 125 Å, 2.7 μm particles). A slurry was made with MeOH (100 mg/mL) and a 200-μm ID capillary column (300 mm long) was packed using steel based union and frits from VICI-Valco. The separation conditions were previously described by Gargano et al. (40). Briefly, the sample was loaded on a C4 trap-column (5 mm × 300 μm ID, 5 μm, 300 Å, Thermo Fisher Scientific), at 15 μL/min for 3 min using the loading pump and a mobile phase of 2% ACN in water with 0.1% TFA. The separations were achieved using mobiles phases that consisted, respectively, of 98% ACN, 2% water, 0.1% TFA for solvent A and 10% 2-propanol, 2% ACN, 0.1% TFA for solvent B, at a flow rate of 4 μL/min. The gradient program had an initial hold at 10% of B for 1 min (min 3 to 4), followed by a 1 min step from 10 till 25% B (min 4 to 5), from 25 to 33% B (min 5 and 6) follow by a linear gradient from 33% B to 36% B in 19 min (min 6 to 25). The solvent composition was then increased in 1 min (min 25–26) to 55% B and in 3 min to 90% B (min 26–29), held at 90% for 2.5 min (min 29 – 31.5), and then washed using a program going from 90% B to 20%, 20% to 90% B and 90% B to 10% B in 1 min steps (min 31.5–32.5 90 to 20% B, min 32.5 – 33.5 20 to 90% B, min 33.5 – 34.5 90 to 20% B, min 34.5 – 35.5 20% – 90% B and min 35.5 – 36.5 90–10% B). After this the column was equilibrated for 5 min.

A capillary (750 mm, 20 μm ID) was used to couple the HILIC column to the CaptiveSpray electrospray ionization (ESI) source of a quadrupole time-of-flight (q-TOF) MS instrument (Maxis HD, Bruker, Bremen, Germany). To reduce TFA ionization suppression, we used a CaptiveSpray nanobooster. This ESI source allows the introduction of dopant enriched nitrogen, here used in combination with a solution containing ACN and 0.5% propionic acid. During operation, the level of dopant solvent was kept above 200 mL. It was then subjected to a pressure of 0.35 bar as well as a dry gas of nitrogen at 3 L/min and at 240°C. The mass spectrometer was operated in positive-ion mode with an electrospray voltage of 1.3 kV. The quadrupole ion and collision cell energies were 6 and 12 eV, respectively. The collision cell RF was 2500 Vpp. The in-source CID (isCID) was set to 80 eV. The funnel RF was 400 Vpp, and the multipole RF was 600 Vpp. The transfer and prepulse storage times were set at 150.0 and 20.0 μs, respectively. The monitored mass range was 400- 4000 m/z. The MS acquisition rates were set to 0.5 Hz.

### Sheathless CE-MS Analysis

Sheathless integrated capillary electrophoresis electrospray ionization was performed on a CESI 8000 system (Sciex, Framingham, MA). The separations were performed using a polyethylenimine (PEI)-coated capillary (91 cm × 30 μm ID). Capillaries were in-house coated with PEI following the protocol described by Sciex (41). The background electrolyte (BGE) and conductive liquid was composed of a solution of 20% AA and 10% MeOH. Before each run, the capillary was simply flushed for 4 min at 100 psi with the BGE. Separation was performed by applying −20 kV at 20°C. Samples were hydrodynamically injected by applying 2.5 psi for 15 s. Before injection, the samples were re-suspended in 10% AA to obtain a concentration of 1 mg/mL. These were then diluted in an equivalent volume of water in order to have 0.5 mg/mL of sample.

The CE system was coupled to a q-TOF MS instrument (Impact, Bruker, Bremen, Germany) via a sheathless CE-ESI source (nano-ESI). The mass spectrometer was operated in positive ionization mode using the following parameters: capillary voltage 1.2 kV, drying gas temperature 100°C, drying gas flow rate 1.2 L/min at a pressure of 0.2 bar. The quadrupole ion and collision cell energy were 5 eV in both cases and the in-source CID (isCID) was 0 eV. The collision cell RF was set at 1500 Vpp. The funnels RF 1 and 2 were 190 Vpp and 600 Vpp, respectively. The hexapole RF was 210 Vpp. The transfer time was 90 μs and the prepulse storage time was 20 μs. MS data was acquired between m/z 500 and 3000 with a spectral acquisition rate of 1 Hz.

### Data Analysis

Data analysis for both CE-MS and HILIC-MS experiments were performed using Compass data analysis (version 5.0, Bruker). Molecular masses were determined using the Maximum Entropy deconvolution algorithm and monoisotopic masses were obtained using the SNAP option of the DataAnalysis software. Extracted-ion chromatograms and electropherograms were obtained with an extraction window of ±0.1 m/z using multiple charge states.

The relative abundances of each glycoform were calculated from the total area of each peak. Each measurement was performed three times in order to get the averages, standard deviations and the coefficients of variation (CVs). Donor 5 was used as a control for all the experiments.

### IgG Glycopeptide Analysis and Data Processing

10 μg of Human plasma IgGs were isolated and analyzed, as described by Falck et al. (42), using 2 μL of FcXL affinity beads (Thermo Fisher Scientific). After elution, antibodies were reduced and alkylated in a 10 μL solution composed of Tris buffer (100 mM, pH 8.5) with 1% SDC, 10 mM TCEP and 40 mM CAA, followed by 5 min shaking at 450 rpm, and an incubation at 95°C for 10 min. Finally, the samples were cleaved overnight at 37°C by addition of 50 μL of 0.4 μg/mL of sequencing grade modified trypsin in 50 mM ABC (pH 8.5). SDC in the samples was then neutralized by 2% FA and after centrifugation the supernatant was collected and diluted 50 times prior to LC-MS analysis.

The RPLC separations were performed on an Ultimate 3000 RSLCnano LC system (Thermo Fisher Scientific) using a Acclaim PepMap 100 C18 column (150 mm x 75 μm ID, 3 μm particles). After injection of 1 μL of the sample onto the column at a flow of 700 nL/min, the separation was achieved with mobile phases composed, respectively, of water with 0.1% FA for A and 95% ACN with 0.1% FA for B. The binary gradient was set up as following: 0–5 min 1% B, linear gradient to 27% B 5–20 min, washing at 70% B 21–23 min, and re-equilibration at 1% B 24–42 min. The LC system was coupled to a q-TOF MS instrument (Maxis, Bruker, Leiderdorp, Netherlands) equipped with a nanobooster and using a nanoESI source. Ionization parameters were as previously reported. Raw LC-MS data were treated with an in-house developed software (LacyTools version 1.1.0) as previously described (42, 43), with the exception of the extraction window which was set at 65 mTh.

### Nomenclature

In this study, we refer to the allotypes with the IMGT allele names, as described by Lefranc and Lefranc (5). Thus, for example, the third allotype of the first subclass of IgG is called IGHG1^∗^03.

The individual glycoforms are represented using the Symbol Nomenclature for Glycans (44). The most common *N*-glycans found in IgG belong to the complex type. Their structure are described by adding galactoses (G0, G1, G2), fucose (F), bisecting GlcNAc (N) or sialic acid (S) to the core structure. As a result, a glycan that presents one galactose and one fucose can be labeled as G1F, and so on.

## Results

### Middle-Up Analysis of Polyclonal IgGs

**FIGURE 1:**
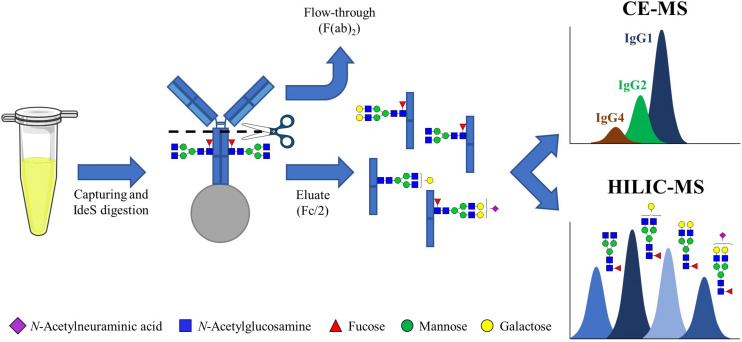
Workflow for the analysis of IgG measuring Fc/2 subunits. IgGs from human plasma and mAbs were captured using C_*H*_3-domain specific beads. The antibodies retained on the beads were then incubated with IdeS, which cleaves IgGs in the C_*H*_2 domain, at 37°C overnight. After removing the F(ab)_2_, in the flow-through, the mixture of single Fc subunits was eluted from the beads and analyzed by HILIC-MS and CE-MS.

To isolate the IgG-Fc subunits from human plasma samples, a C_*H*_2-region digestion was performed after IgG purification. To this end, IgGs were captured using anti-Fc beads, followed by the addition of the enzyme IdeS which is effective for all IgG subclasses. After digestion, the F(ab)_2_ subunits were collected in the flow-through fraction and the IgG Fc subunits were eluted from the beads, enabling the isolation of Fc/2 subunits (single heavy chain Fc) from plasma (Figure 1). Prior to analysis, sample purity, IgG capturing efficiency and IdeS digestion efficiency were determined by non-reducing SDS-PAGE (Supplementary Figure S1).

**FIGURE 2:**
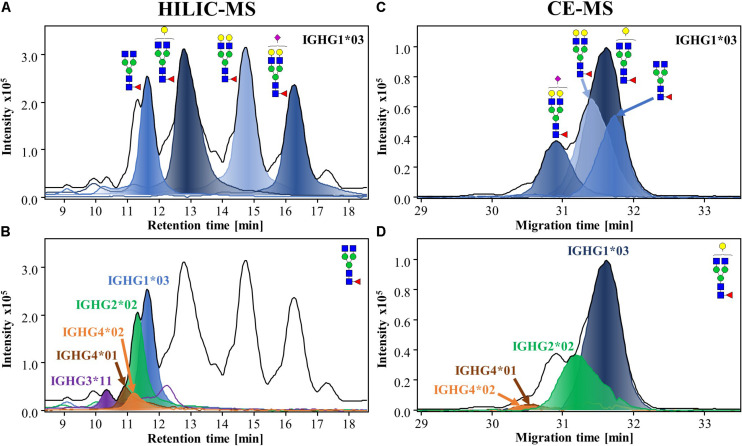
HILIC-MS **(A,B)** and CE-MS **(C,D)** of IgG-Fc/2 proteoforms from human plasma from a single donor (Donor 1). The separation of Fc/2 subunits after IdeS digestion are visible through their base peak chromatograms (BPCs) and base peak electropherograms (BPEs), which are represented by black lines. Extracted-ion chromatograms (EICs) and electropherograms (EIEs), from the same donor, of the different glycoforms (filled lines) of IGHG1*03 **(A,C)** and EICs of G0F **(B)** and EIEs of G1F **(D)** illustrating the resolution of the different subclasses and allotypes (For a better illustration of CE separation, EIEs of G1F were selected). HILIC-MS conditions: injection of 0.083 mg/mL solution. Temperature 50°C. Linear gradient from 10% to 25% B in 1 min, from 25% to 33% B in 1 min, from 33% to 36% B in 19 min, from 36% to 55% B in 1 min and from 55% to 90% B in 3 min. CE-MS conditions: injection of 0.5 mg/mL at 2.5 psi for 15 s. BGE, 20% acetic acid and 10% methanol. Separation voltage and temperature, –20 kV and 20°C, respectively.

The mixture of Fc/2 proteoforms (Mw ∼ 25 kDa) was analyzed by HILIC-MS and CE-MS. The results of the analysis from one of the donors (Donor 1) are shown in Figure 2. HILIC resolves proteoforms according to hydrophilic interactions, causing glycans to have a major impact on the separation. As a result, the resolution of multiple glycoforms was possible, with the four most abundant ones, G0F, G1F, G2F, and G2FS1, eluting within distinct retention time windows. The extracted ion chromatograms (EICs) of the glycoforms from one specific allotype (IGHG1^∗^03) are shown in Figure 2A. The resolution of the different subclasses and allotypes was limited in HILIC, especially for IgG2 and IgG4 which overlap in the chromatogram (Figure 2B). CE separation, on the other hand, provides separation of proteoforms with differences in charge or size. CE provided good separation of IgG2 and IgG4 and certain allotypes as a consequence of their different amino acid sequences (Figure 2D). In addition, CE separation is affected by charge causing sialylated glycoforms to migrate earlier than neutral glycoforms (Figure 2C). HILIC-MS resolved a larger number of species and, therefore, was used for the assignments, while CE-MS was employed for confirmation purposes due to its orthogonality. Both, CE-MS and HILIC-MS showed good repeatability and intermediate precision for the relative abundances of the four majors glycoforms of IgG1 (Supplementary Figures S2A,B). The median CVs for the abundance of all the glycoforms were 3.1% for CE and 2.6% for HILIC-MS.

**FIGURE 3:**
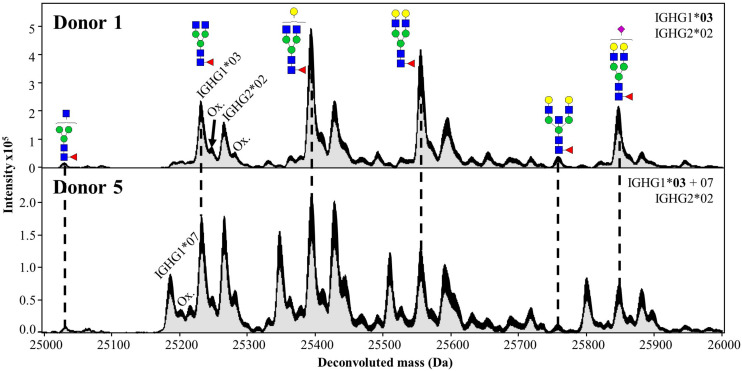
Deconvoluted mass spectra (10–18 min) of Fc/2 from Donors 1 and 5 obtained during HILIC-MS. The Maximum Entropy deconvolution algorithm was used to transform *m/z* values into molecular masses in Daltons (Da). Each annotated glycoform corresponds to IGHG1*03. Additional peaks correspond to the glycoforms of other allotypes as well as their oxidized forms (Ox.). Dotted lines represent the same glycoforms from the two IGHG1*03 of Donor 1 and 5.

**FIGURE 4:**
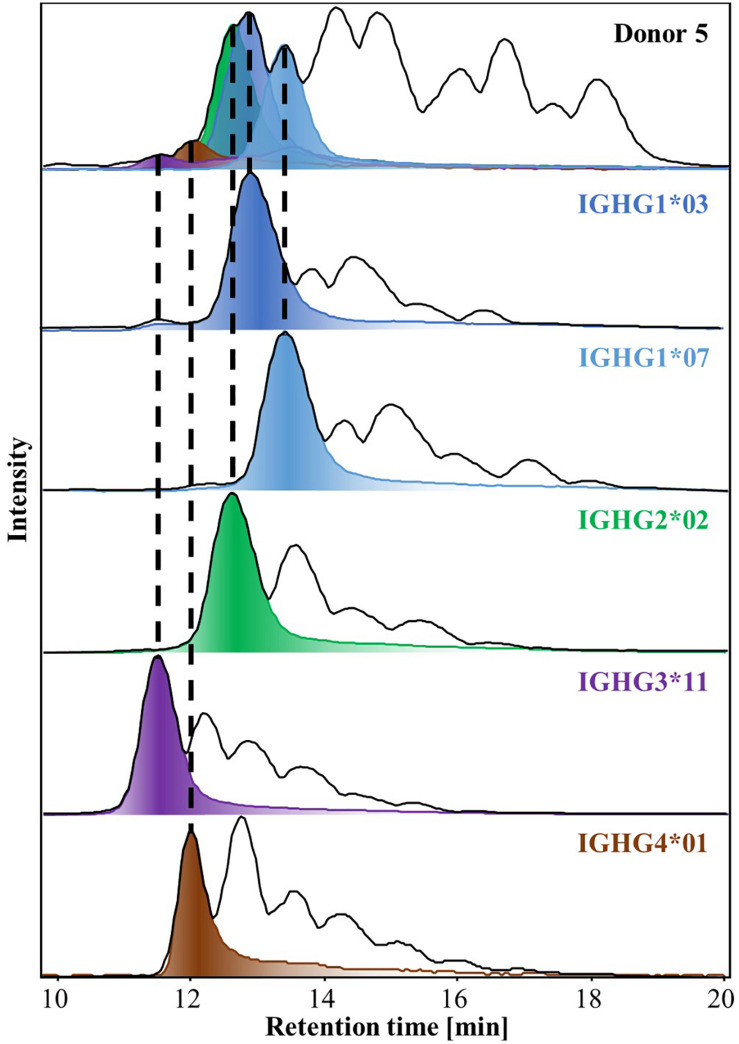
BPCs and EICs of G0F obtained by HILIC-MS of IgG-Fcs isolated from Donor 5 (top trace) and from recombinant IgGs of the relevant allotypes (bottom traces). Dotted lines match the EICs of G0F from Donor 5 to the equivalent EICs of each mAbs allotype, exhibiting their similar retention times. HILIC-MS conditions: injection of 0.083 mg/mL solution. Temperature 50°C. Linear gradient from 10% to 25% B in 1 min, from 25% to 33% B in 1 min, from 33% to 36% B in 19 min, from 36% to 55% B in 1 min and from 55% to 90% B in 3 min.

Upon spectral deconvolution of the mass spectra acquired during HILIC-MS (Figure 3), all Fc/2 detected appeared to be lysine-clipped at their C-terminus, non-reduced (i.e., intact disulfide bridges), and carrying different glycoforms. Monoisotopic masses were obtained using the SNAP algorithm. Mass errors (between −10 and 10 ppm) were low for most of the allotypes (Table 1). To reinforce our assignments, each of the 27 known allotypes was recombinantly produced and analyzed by HILIC-MS. The retention times of the allotypes found in each donor were then compared to the ones obtained for the monoclonal allotypes. EICs of G0F from all donors nicely matched to EICs of G0F from individual allotypes (Figure 4), with average deviations from the control sample ranging from 0.03 min for IGHG2^∗^02 to −0.14 min for IGHG4^∗^01 (Supplementary Table S1).

**TABLE 1:** Allotypes identified by HILIC-MS for each of the analyzed donors.

Fc/2 monoisotopic masses (non-reduced + Lysine clipped) for G0F						
Donor	Sex	Age (years)	Allotypes	Experimental mass (Da)	Theoretical mass (Da)	Mass error (ppm)
1	Male	59	IGHG1*03	25216.71	25216.43	11.01
			IGHG2*02	25250.55	25250.33	8.63
			IGHG3*11/12	25236.41	25236.44	−1.18
			IGHG4*01	25200.30	25200.34	−1.52
			IGHG4*02	25186.36	25186.32	1.37
2	Male	47	IGHG1*01	25184.45	25184.46	−0.57
			IGHG1*03	25232.02^#^	25231.99^#^	1.44
			IGHG2*01	25233.96^#^	25233.98^#^	−0.94
			IGHG2*06	25234.50	25234.35	6.03
			IGHG3*01	25252.37	25252.44	−2.62
			IGHG3*11/12	25236.37	25236.44	−2.62
			IGHG4*01	25200.29	25200.34	−2.01
3	Female	27	IGHG1*03	25216.20	25216.43	−9.25
			IGHG2*02	25250.11	25250.33	−8.65
			IGHG3*11/12	25236.36	25236.44	−3.17
			IGHG4*01	25200.17	25200.34	−6.50
			IGHG4*02	25186.13	25186.32	−7.50
4	Female	66	IGHG1*01	25184.68	25184.46	8.58
			IGHG1*07	25170.53	25170.44	3.65
			IGHG2*01	25218.38	25218.36	0.73
			IGHG3*14	25312.46	25311.47	39.03
			IGHG4*01	25201.32	25200.34	39.08
			IGHG4*03	25172.43	25172.33	4.03
5	Female	52	IGHG1*03	25217.65	25216.43	48.29
			IGHG1*07	25170.61	25170.44	6.55
			IGHG2*02	25250.38	25250.33	2.05
			IGHG3*11/12	25236.42	25236.44	−0.63
			IGHG4*01	25200.30	25200.34	−1.39

*Observed and theoretical monoisotopic masses and experimental errors depicted, correspond to non-reduced G0F Fc/2 subunits after lysine clipping. All masses correspond to monoisotopic masses (Rs ∼ 80000 for HILIC-MS analyses) except (^#^) where average masses were used as measurement were performed with CE-MS (Rs ∼ 40000) (sex and age, at the time of plasma collection, are indicated for each donor).*

### Subclass and Allotype-Specific Analysis of Fc Subunits of Individual Donors

HILIC-MS of plasma of five independent donors yielded between 4 to 12 peaks of high intensity in the resulting base peak chromatograms (BPCs) (Supplementary Figure S3). These peaks corresponded to the four most abundant glycoforms of the present IgG1 and IgG2 allotypes, which are the two most abundant subclasses. For instance, Donor 5 in Supplementary Figure S3J carries IGHG1^∗^03, IGHG1^∗^07 and IGHG2^∗^02. The three allotypes were partially separated, producing three distinguishable peaks for each glycoform (Supplementary Figure S3I). For Donor 2, the allotypes IGHG1^∗^03 and IGHG2^∗^01 were observed which exhibit virtually the same retention times in HILIC and very similar *m/z* values (for charge state + 15, *m/z* 1683.1587 and 1683.2235, respectively) (Supplementary Figure S3D). While it was not possible to resolve these allotypes in HILIC, they showed some degree of separation in CE (Supplementary Figure S4) permitting their assignment.

**FIGURE 5:**
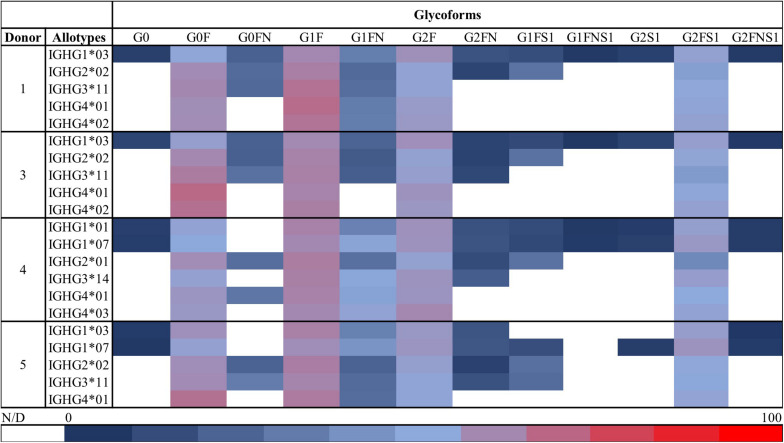
Overview of the relative abundances of the glycoforms detected by HILIC-MS for all the allotypes in each of the five assessed donors. Each row corresponds to the relative abundances of the glycoforms found for one allotype of one donor. Relative abundances were obtained using the total area of each peak from all the present glycoforms within one donor. Red indicates a high relative abundance, blue a low relative abundance and white represents glycoforms that were not observed or for which relative quantitation was not possible. The color scale is shown at the bottom of the figure. Glycoforms from Donor 2 were quantified with both CE and HILIC and thus not shown here. HILIC-MS conditions: injection of 0.083 mg/mL solution. Temperature 50°C. Linear gradient from 10% to 25% B in 1 min, from 25% to 33% B in 1 min, from 33% to 36% B in 19 min, from 36% to 55% B in 1 min and from 55% to 90% B in 3 min.

Table 1 summarizes all the IgG allotypes found for the five donors included in this study. Between 5 and 7 allotypes were identified for each donor. Subsequently, the different glycoforms of each specific Fc/2-allotype were investigated. The relative abundances of all glycoforms observed by HILIC-MS for Donors 1 and 3–5 are shown in Figure 5. For the subclasses of lower abundance (1 ≥ 2 ≥ 3/4, Supplementary Table S2) a lower number of glycoforms was detected. Nevertheless, the four most abundant glycoforms were observable for all allotypes, with the exception of IGHG3^∗^01 and IGHG3^∗^11/12 from Donor 2. In order to support the results obtained with our approach, the glycosylation levels determined for IgG1 were compared with those obtained with a well-established glycopeptide protocol (42) using RPLC-MS (Supplementary Figure S2C). In RPLC-MS, glycopeptides can only reflect the total IgG1 as the peptide obtained for the different allotypes have the same amino acid sequence. Thus, the abundances of the two allotypes IGHG1^∗^03 and IGHG1^∗^07 obtained by HILIC-MS and CE-MS were determined individually and the obtained values were summed for the calculation of relative abundances and comparison to the glycopeptide data. Compared to RPLC-MS, the glycoforms determined by HILIC-MS and CE-MS showed similar relative abundances (e.g., G0F relative abundance is 21.7% in HILIC, 23.6% in CE and 25.3% in RPLC).

**FIGURE 6:**
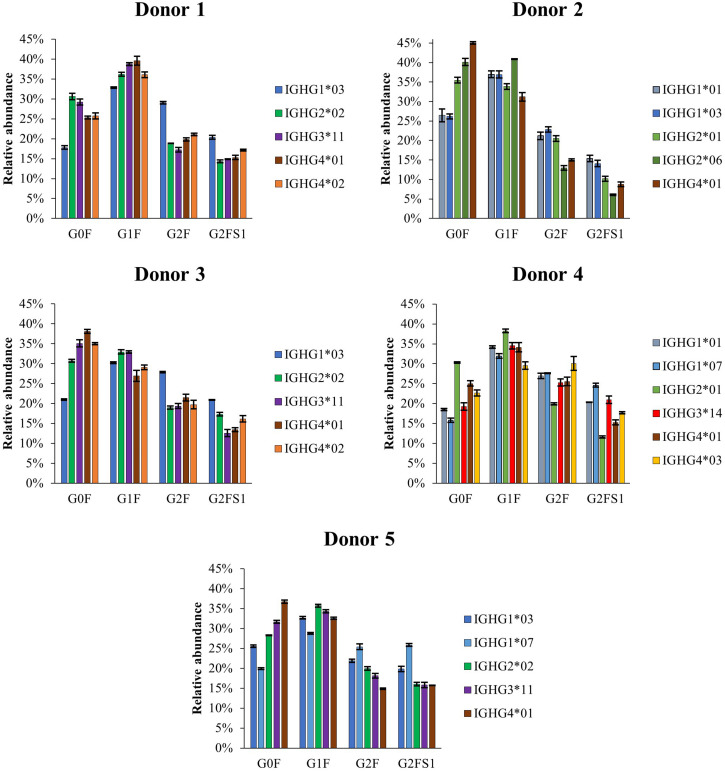
Normalized relative abundances of the four most abundant glycoforms of the allotypes identified by HILIC-MS for each of the analyzed donors. The normalization was performed based on the relative abundances of the four major glycoforms (G0F, G1F, G2F, and G2FS1) instead of the total area of all the glycoforms. HILIC-MS conditions: injection of 0.083 mg/mL solution. Temperature 50°C. Linear gradient from 10% to 25% B in 1 min, from 25% to 33% B in 1 min, from 33% to 36% B in 19 min, from 36% to 55% B in 1 min and from 55% to 90% B in 3 min.

To compare the glycosylation patterns of subclasses and allotypes, the normalized relative abundances of G0F, G1F, G2F and G2FS1 glycoforms measured for each allotype in the donors were calculated (Figure 6). Most abundances were quantified by HILIC-MS, except for IGHG1^∗^03 and IGHG2^∗^06 from Donor 2; the ratio of these two species as observed in CE-MS were used to allow HILIC-MS quantification. The measured abundances demonstrate a low glycoform diversity among allotypes of the same subclass, but show larger glycosylation differences between subclasses.

In addition, oxidized variants were observed for each glycoform of IgG1 and IgG2 allotypes. As shown in Supplementary Figure S5, all the donors presented around 20–30% oxidation, with a higher degree of oxidation for IgG2 allotypes. However, oxidation did not have a major impact on the glycosylation profiles (Supplementary Figure S6). To determine whether oxidation is biologically relevant or an artifact introduced during the analysis or storage, two HILIC-MS analyses of plasma of Donor 3 were performed 9 months apart. An increase in oxidation of ∼15% was observed (Supplementary Figures S7, S8) indicating that oxidation occurred during storage of the sample.

## Discussion

### Development of a Middle-Up MS-Based Strategy for the Analysis of Polyclonal IgG-Fc

In order to solve the challenge of analyzing polyclonal IgGs while getting a comprehensive overview of the glycosylation and other PTMs of each specific allotype, we developed an MS-based middle-up approach. This approach allowed the isolation of Fc/2 subunits from plasma of healthy donors after selective IgG trapping and on-bead digestion with IdeS. However, the resulting Fc subunits comprise diverse subclasses, allotypes and PTMs of interest with very similar molecular masses. After limited proteolytic cleavage, certain allotypes within one subclass even present identical mass (e.g., IGHG2^∗^01 and IGHG2^∗^04) or differ only slightly (e.g., 1 Da for IGHG3^∗^03 and IGHG3^∗^11/12). This also occurs for allotypes belonging to different subclasses (e.g., a difference of 1.86 Da for IGHG1^∗^01 and IGHG4^∗^02). Moreover, PTMs such as citrullination and deamidation, which bring only a subtle change in the molecular mass (<1 Da), may further increase the complexity causing the overall characterization by stand-alone MS to be very challenging. Hence, we explored two orthogonal separation techniques, HILIC and CE, for the separation of the Fc/2 subunits prior to MS detection.

HILIC analysis allowed the resolution of all the allotypes present for each donor and showed particularly strong in providing glycosylation profiles. CE-MS showed a lower resolving capacity in comparison with HILIC-MS, but permitted separation of IgG1, IgG2, and IgG4 subclasses. Nonetheless, the orthogonality of the two techniques was key for the assignment of some allotypes, especially in the case of Donor 2, where IGHG1^∗^03 and IGHG2^∗^01 (Δ = 1.93 Da) presented the same retention times and similar *m/z* values in HILIC-MS, but were partially resolved in CE-MS. Even for a complex donor such as Donor 2, who is heterozygous for IgG1-3, more than 41 individual proteoforms could be determined. For less complex donors the number of observed proteoforms ranged from 35 to 47 (Figure 5).

The use of high resolution MS allowed to identify the specific allotypes present in each donor with high accuracy (0.5–11.0 ppm) (Table 1). Only few allotypes, IGHG1^∗^03 of Donor 5 as well as IGHG3^∗^14 and IGHG4^∗^01 from Donor 4, exhibited a higher error. Comparison with the theoretical mass spectra revealed good matches of the isotopic distribution for IGHG3^∗^14 and IGHG4^∗^01 (Supplementary Figure S9). The high ppm error in the monoisotopic masses of these two allotypes could be explained by the use of the SNAP algorithm which is highly sensitive to overlapping species. Only for IGHG1^∗^03, a slight discrepancy was observed between the observed and the calculated mass spectra. The MS analysis also revealed oxidation of IgG1 and IgG2. The oxidation increased with the storage time suggesting an artifact rather than biological variation. The oxidation levels were higher for IgG2 allotypes which can be explained by the presence of more methionines in IgG2 than in IgG1.

The proposed middle-up approach provides the capability to study the glycosylation in a subclass and allotype specific manner. State-of-the-art glycopeptide workflows for studying IgG glycosylation do not allow resolution of IgG3, whose tryptic peptide is identical or isomeric to the one of IgG2 or IgG4 depending on the allotype (23, 45). Therefore, IgG3 glycosylation was previously only accessible via elaborate targeted purifications (45, 46). The relative abundances for glycoforms obtained with both middle-up and glycopeptide approaches were similar (Supplementary Figure S2C) demonstrating that the proposed approach permits relative quantitation of subclass-specific IgG glycosylation. Only the levels of sialylation seem slightly higher for CE-MS and HILIC-MS results as compared to RPLC-MS (∼2 and 4% lower). Presence of sialic acids may result in relatively lower ionization of glycopeptides (47), whereas this effect may be less pronounced for the ionization of the much larger Fc/2 subunits. The characterization of IgG-Fc sialylation is important because of its anti-inflammatory effects (48). On the other hand, analysis of glycopeptides provides a higher sensitivity as compared to the analysis of intact Fc subunits, resulting in considerably higher numbers of glycoforms detected (49).

### Allotype-Specific Characterization of Glycosylation

In this study, we assessed the glycosylation patterns of five independent donors. We included donors with different age and sex (Table 1), as glycosylation differences depending on these factors are well-documented (50–52). In Table 1, we reported all the allotypes found in the five donors. Donors comprised a total of 5 to 7 allotypes and presented a high variability of IgG allotypes within the same population (Caucasoid population). All the donors present heterozygosis in at least one of the subclasses resulting in a total of 12 allotypes identified. IGHG1^∗^03, IGHG2^∗^02 and IGHG3^∗^11/12 were the most persistent allotypes from the first, second and third subclasses, as expected for Caucasoid populations (5). We also observed that even if IGHG3^∗^11/12 were predominant, they were not the only IgG3 allotypes present. In Europe, it is often assumed for IgG glycopeptides studies that most of the samples originate from Caucasian people with rather monogenic IgG3 allotypes (i.e., IGHG3^∗^11 or IGHG3^∗^12), which have the same amino acid sequence as IgG2. Therefore, the resolution of these two subclasses cannot be achieved with standard glycopeptide methods, emphasizing the importance of our approach which allows resolving subclasses and avoid ambiguities. Regarding IgG4, all the donors presented IGHG4^∗^01 and other allotypes were only observed in heterozygotic donors (Donor 1, 3 and 4).

We determined for each donor the different allotypes and the relative abundances of their respective glycoforms. Figure 5 shows the overview of these relative abundances determined for each specific subclass and allotype present (i.e., a donor-specific Fc-proteoform map). For all observed allotypes, the most abundant glycoforms corresponded to diantennary glycans containing from 0 to 2 galactoses, a core fucose and no or one sialic acid. This is in line with the literature (53, 54) and was confirmed by an established bottom-up glycoproteomics method. For high abundant allotypes, afucosylated and bisected species were also detected. As expected, the glycoform patterns show a donor-specific dependence. As an example, Donor 3 shows a higher agalactosylation while Donor 4 appears to have a slightly higher galactosylation. Most donors exhibit a higher abundance of G0F, G1F, with the exception of Donor 4 who has a more distributed glycosylation profile. This phenomenon cannot be explained by the advanced age of Donor 4 as galactosylation generally decreases with age (52).

For comparing the glycosylation profiles between subclasses and allotypes, we focused on the four most abundant glycoforms (Figure 6). Different subclasses showed clear differences in the glycosylation patterns. IgG1 shows a higher galactosylation and sialylation than the other three subclasses, and IgG4 has a higher abundance of G0F in Donors 2, 3, and 5. The differences in glycosylation are especially apparent for IgG2, which exhibits lower level of galactosylation and sialylation as reported before by Plomp et al. (23). This highlight again the importance of distinguish between subclasses when studying glycosylation changes to avoid bias in result interpretation. Regarding different allotypes, there was no obvious difference between the glycosylation profiles of two allotypes from the same subclass within donors. Between donors, however, the IGHG3^∗^14 allotype seems to exhibit a higher degree of galactosylation and sialylation in comparison with IGHG3^∗^11/12 allotypes. This observation, was also previously described for mAbs by de Taeye et al. (11). So far, we only observed 12 of the 27 existing allotypes, with few combinations of them. We would need several donors from different ethnicities in order to validate or exclude an allotype-specific glycosylation hypothesis and the influence of the amino acid sequence on the glycosylation.

## Conclusion

We developed a new middle-up MS-based approach which allows for the first time to study glycosylation profiles of polyclonal IgG in a subclass and allotype-specific manner. In a single analysis, the allotypes present in a donor and their glycosylation patterns can be obtained and the relative abundance of different subclasses estimated. Furthermore, the proposed approach has the ability to determine additional co-occurring modifications in the Fc subunits as illustrated for oxidation. Future applications of this method could prove themselves useful regarding a more accurate study of disease-related glycosylation changes and in the study of subclass or allotype-specific diseases (e.g., IgG4 autoimmune diseases). Our results showed a clear variability of the glycosylation profiles for independent donors. While these differences seem to be donor and subclass associated, no evidence for allotype-dependent glycosylation was observed between the allotypes detected in each donor. Next steps will focus on the analysis of a larger number of samples from different populations to detect all the possible allotypes and study potential differences in their glycosylation patterns.

## Data Availability Statement

The datasets presented in this study can be found in the online repository: https://doi.org/doi:10.25345/C5K11H, via accession number MSV000085388.

## Ethics Statement

Plasma samples were collected from five different healthy donors in accordance with Dutch regulations and after approval from the Sanquin Ethical Advisory Board in accordance with the Declaration of Helsinki.

## Author Contributions

TS performed the sample preparation. ST prepared the monoclonal standard IgG allotypes. TS and AG carried out the HILIC-MS analysis. TS and ED-V performed the CE-MS analysis and drafted the manuscript. TS processed and analyzed the data. DF supported the bottom-up glycoproteomics experiments. AG, DF, ST, TR, GV, GS, and MW reviewed this manuscript. All authors contributed to the article and approved the submitted version.

## Conflict of Interest

The authors declare that the research was conducted in the absence of any commercial or financial relationships that could be construed as a potential conflict of interest.
