# Supplementary material for: MS-Based Allotype-Specific Analysis of Polyclonal IgG-Fc N-Glycosylation
Source: Front Immunol. 2020 Aug 21;11:2049. doi: 10.3389/fimmu.2020.02049 (PMC7472933; doi:10.3389/fimmu.2020.02049)
Supplement: Supplementary file 1 [file Data_Sheet_1.docx]

***Supplementary Material***

**SUPPLEMENTARY FIGURES**

**
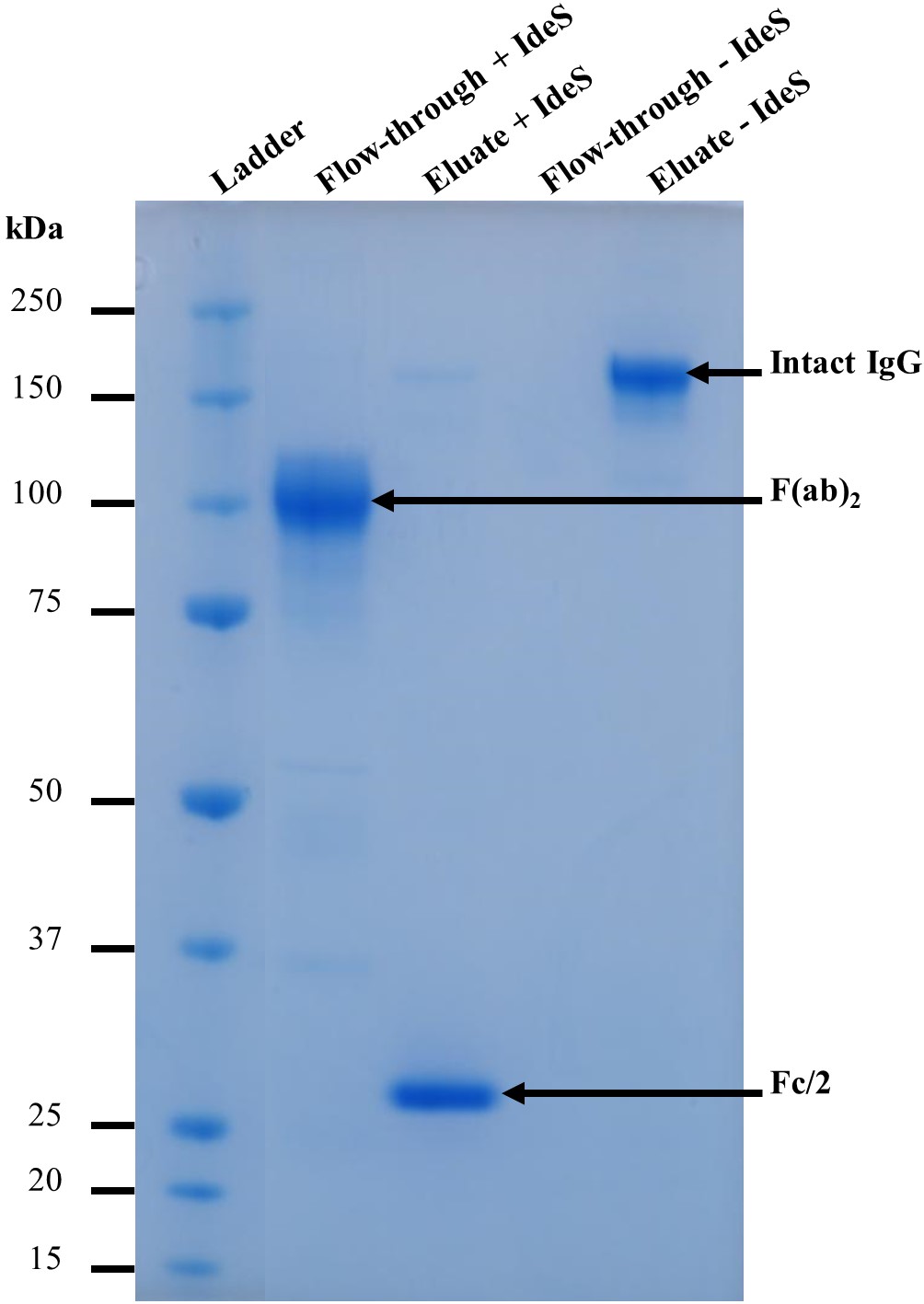
**

**Figure S1**. Non-reducing SDS-PAGE of IgGs from Donor 5 after sample preparation for middle-up analysis. Lane 1 was loaded with a protein ladder. Other lanes were loaded with 5 µg of protein after denaturation at 60°C for 5 min. Lanes 2 and 3 are respectively the flow-through (F(ab)_2_) and the eluate from the beads (Fc/2) after IdeS digestion, while lanes 4 and 5 show the same fractions without adding enzyme, respectively. The separation was performed using a 4-12% Bis-Tris gel at 200 V for 55 min. The gel was then stained with Coomassie blue and visualized under trans-illumination. The assessment of the digestion and capturing efficacies are represented by lanes 2, 3 and 3,4, respectively.


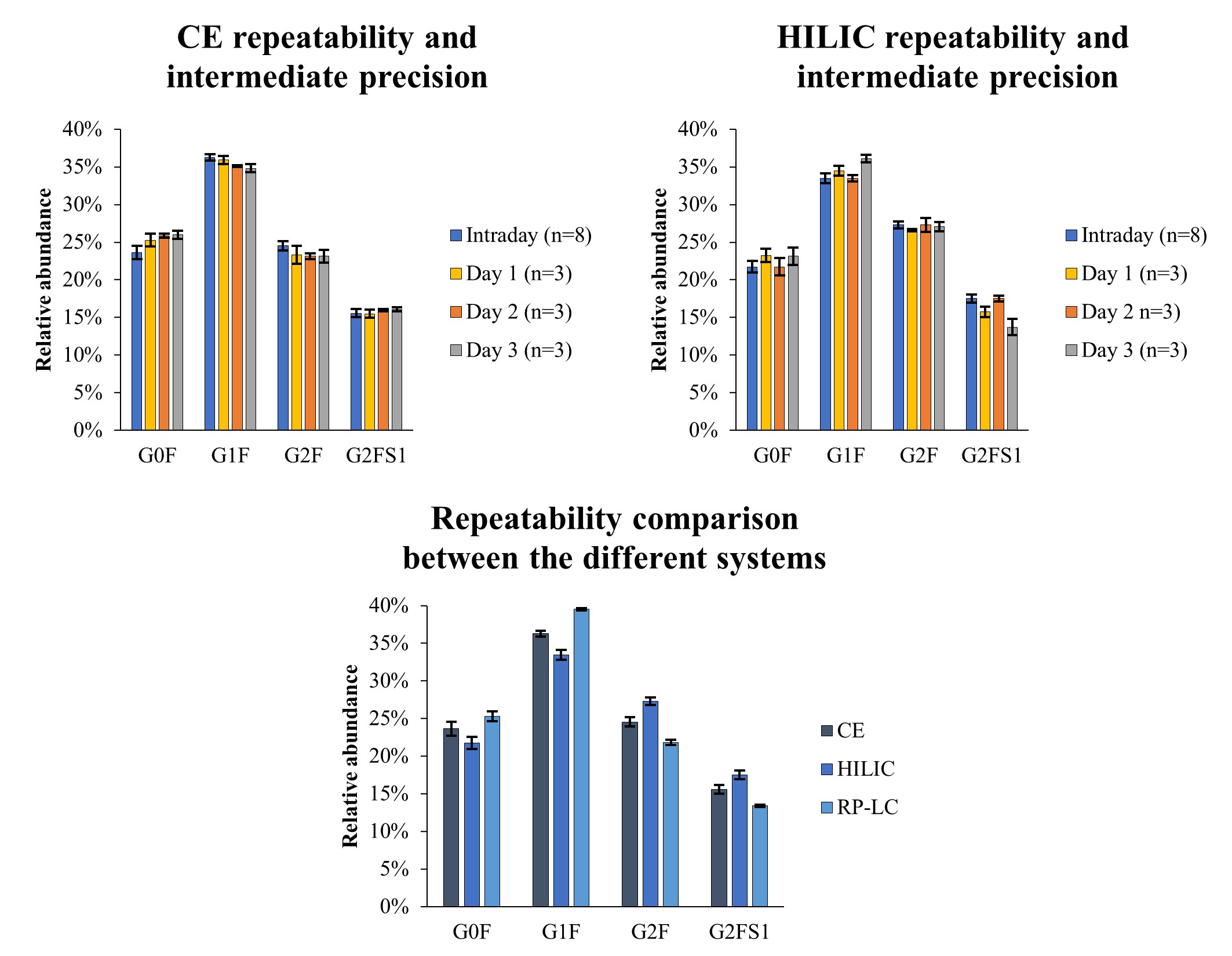


**C**

**A**

**B**

**Figure S2.** Relative abundances of the four major glycoforms of IgG1 (IGHG1*03 and IGHG1*07) present in the control sample as obtained upon CE-MS **(A)** and HILIC-MS **(B)** analysis. Relative abundances were calculated using the total area from EIC of each glycoform and each analysis was performed in triplicate to obtain the standard deviations (SD). Intra- and inter-day repeatability tests were performed with the inter-day repeatability investigated among three days. **(C)** Comparison of the different levels of glycosylation as observed with CE-MS and HILIC-MS for IgG1 Fc subunits with the levels observed by RP-LC-MS for IgG1 glycopeptides after normalization, obtained from the total area of the four major glycoforms (CVs from 0.32% to 3.79%). HILIC-MS conditions: injection of 0.083 mg/mL solution. Temperature 50°C. Linear gradient from 10% to 25% B in 1 min, from 25% to 33% B in 1 min, from 33% to 36% B in 19 min, from 36% to 55% B in 1 min and from 55% to 90% B in 3 min. CE-MS conditions: injection of 0.5 mg/mL at 2.5 psi for 15 s. BGE, 20% acetic acid and 10% methanol. Separation voltage and temperature, -20 kV and 20 ºC, respectively. RP-LC-MS conditions: injection of 0.13 mg/mL solution. Linear gradient from 1% to 27% B for 15 min.


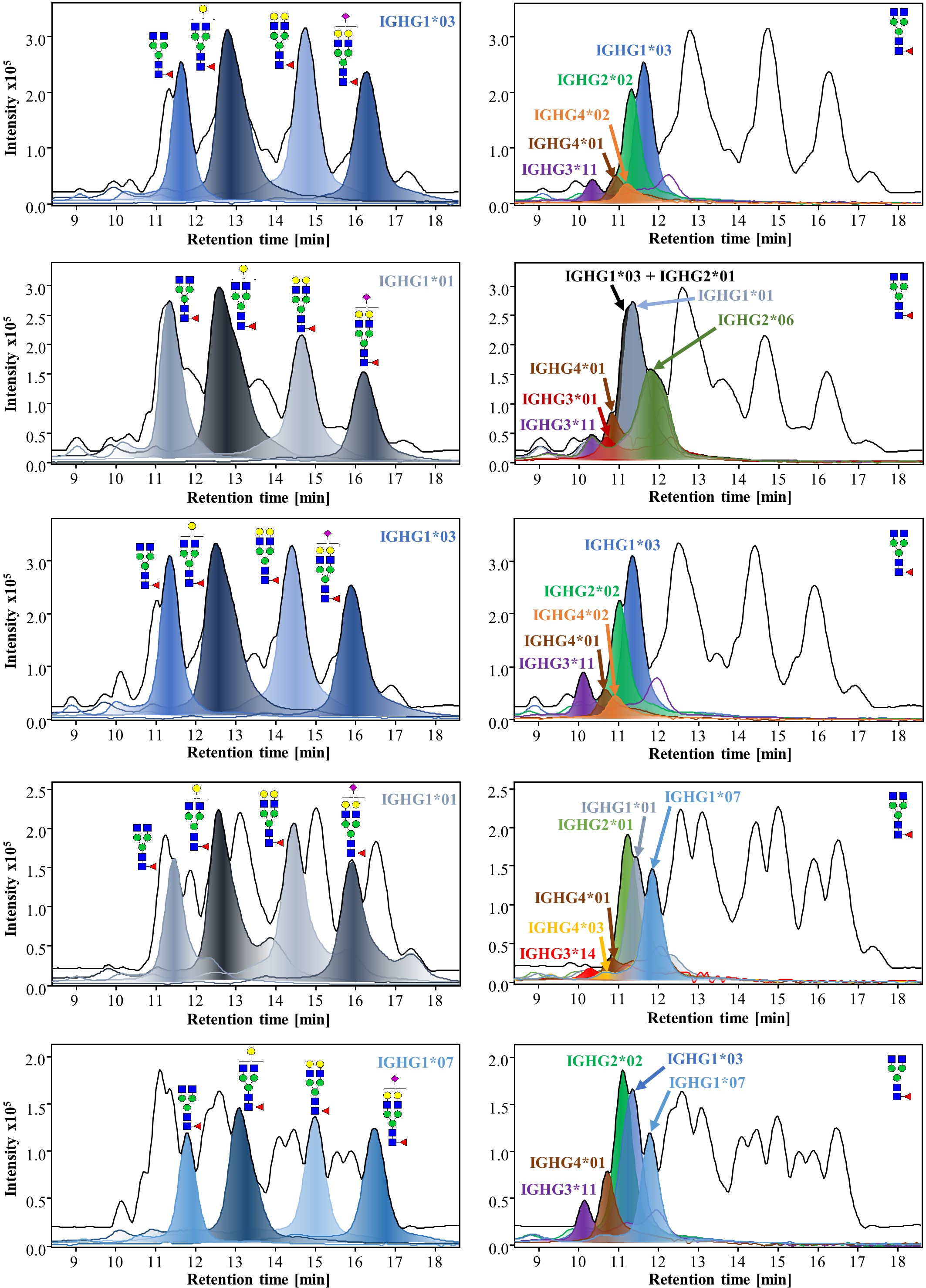


**J**

**I**

**H**

**G**

**F**

**E**

**D**

**C**

**B**

**A**

**Figure S3.** BPCs and EICs obtained with HILIC-MS for Fc/2 subunits from plasma of Donors 1 **(A, B)**, 2 **(C, D)**, 3, **(E, F)**, 4 **(G, H)** and 5 **(I, J)** exhibiting different allotypes and glycoforms. EICs of IgG1 glycoforms illustrating the different elution of the IGHG1*01, IGHG1*03 and IGHG1*07 allotypes **(A, C, E, G, I)**. EICs of G0F for all subclasses and allotypes observed in the different donors within their respective BPCs **(B, D, F, H, J)**. HILIC-MS conditions: injection of 0.083 mg/mL solution. Temperature 50°C. Linear gradient from 10% to 25% B in 1 min, from 25% to 33% B in 1 min, from 33% to 36% B in 19 min, from 36% to 55% B in 1 min and from 55% to 90% B in 3 min.


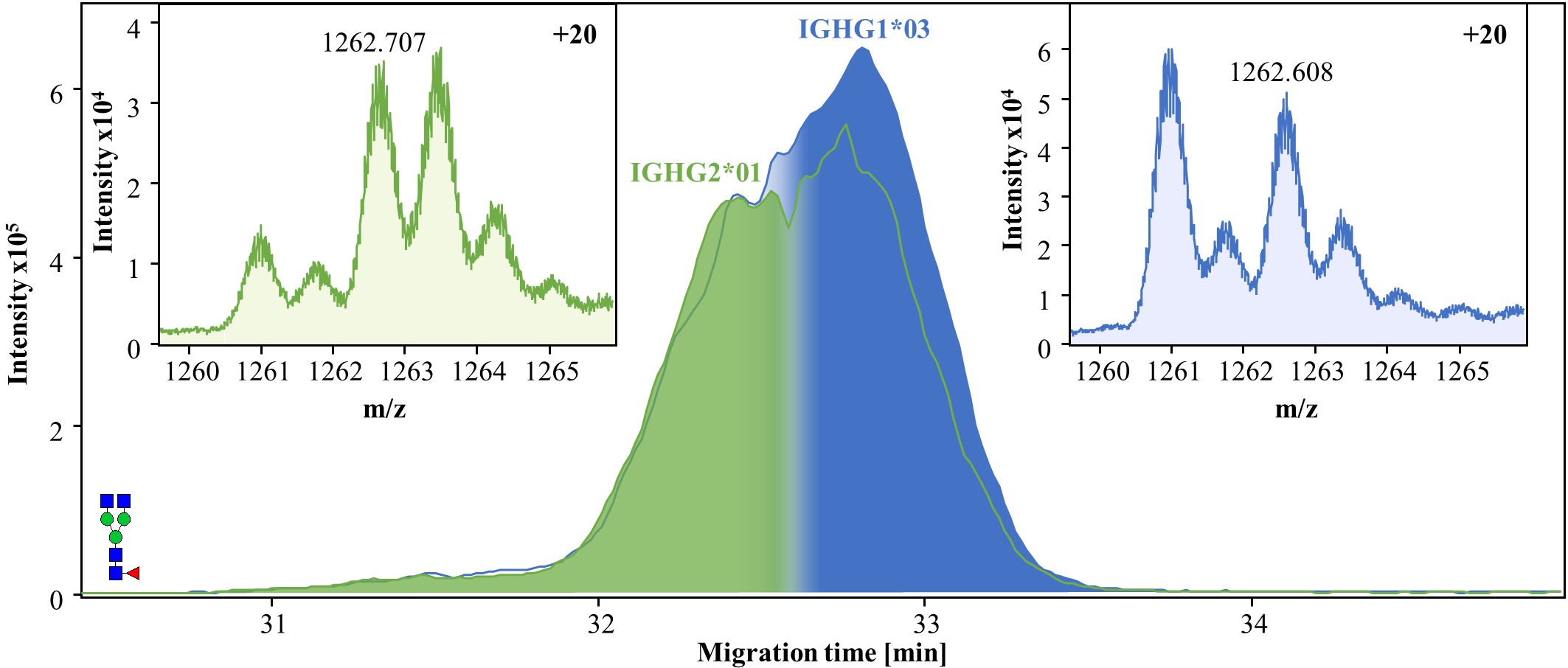


**Figure S4.** Partial separation of IGHG1*03 and IGHG2*01 from Donor 2 obtained by sheathless CE-MS after IdeS digestion of polyclonal IgGs. EIEs of G0F for all the combined charge states ±0.1 m/z are demonstrating the difference in migration for the two allotypes. Mass spectra of charge state +20 show the observed m/z values for IGHG2*01 (theoretical, 1262.708 *m/z*) in green and IGHG1*03 (theoretical, 1262.608 *m/z*) in blue. CE-MS conditions: injection of 0.5 mg/mL at 2.5 psi for 15 s. BGE, 20% acetic acid and 10% methanol. Separation voltage and temperature, -20 kV and 20 ºC, respectively.


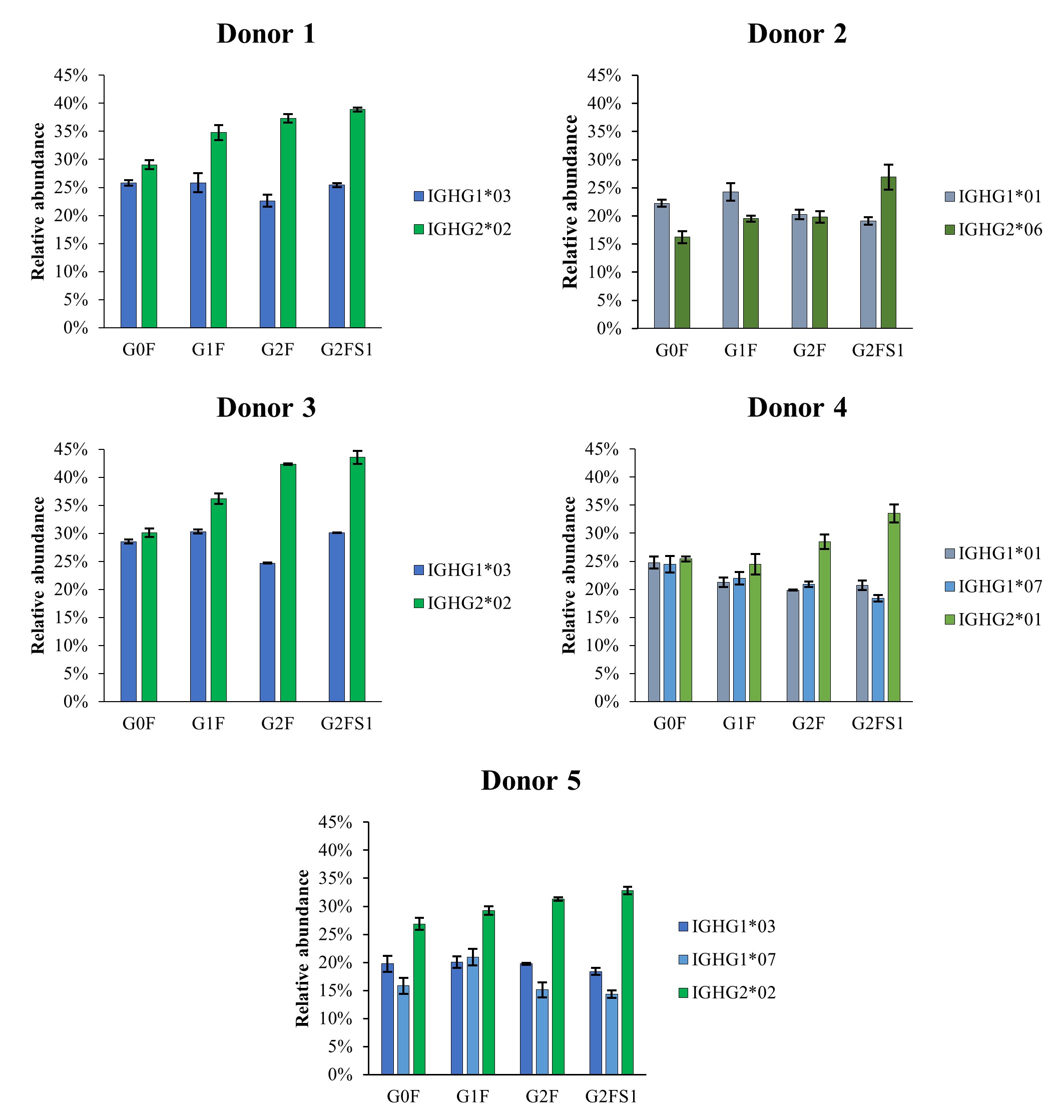


**Figure S5.** Relative abundances of the four most abundant oxidized glycoforms of IgG1 and IgG2 identified by HILIC-MS for each of the analyzed donors. The relative abundances reflect the percentage of oxidized glycoform per glycoform (oxidized vs non-oxidized). The total area of each oxidized and non-oxidized peak was used to calculate the different relative abundances. SDs were determined based on the three replicates of each analysis.


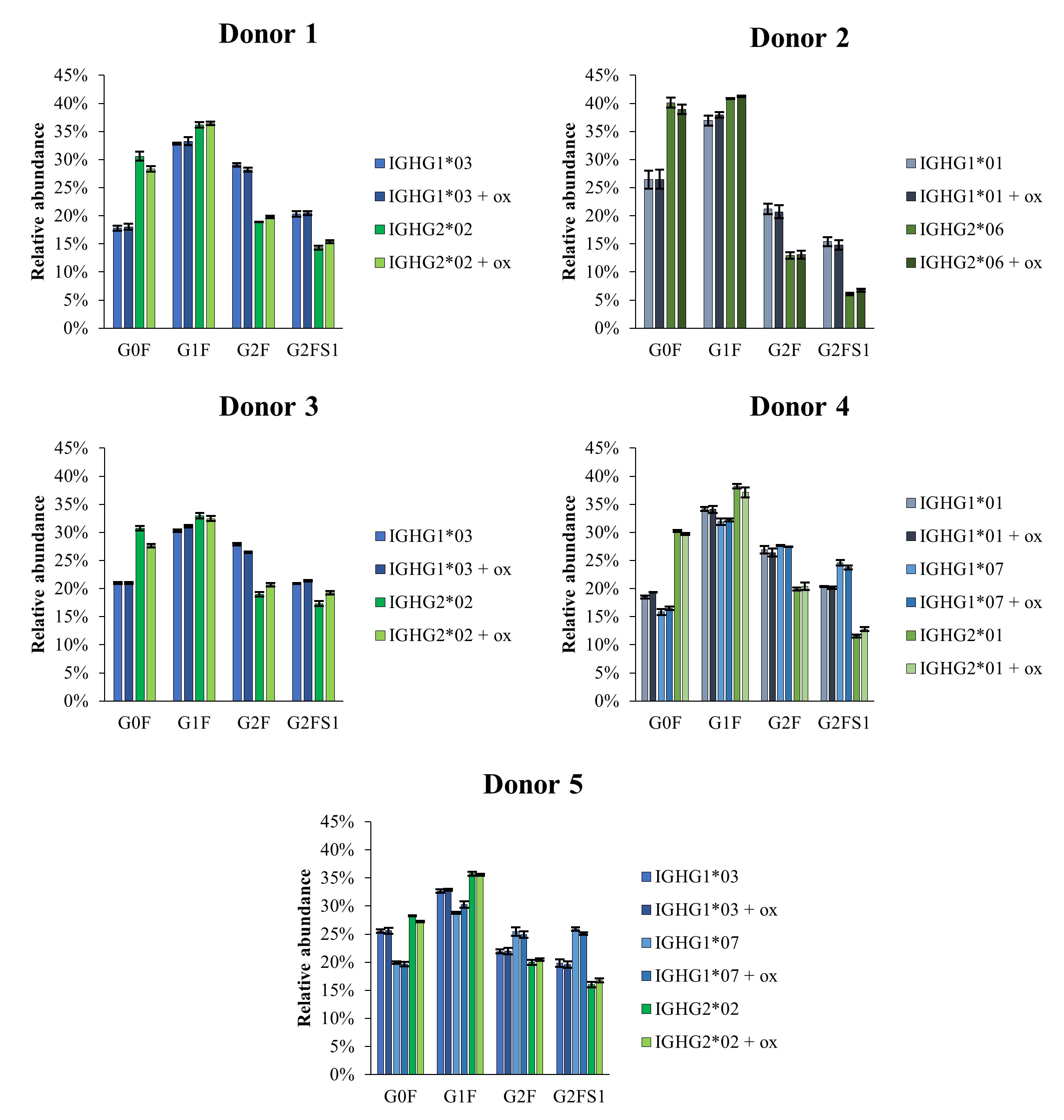


**Figure S6.** Relative abundances of the four most abundant glycoforms of IgG1 and IgG2 identified by HILIC-MS for each of the analyzed donors with and without correction for oxidation (ox). The correction was made by adding the areas of oxidized glycoforms to the total area per allotype (oxidized and non-oxidized glycoforms). SDs were determined based on the three replicates of each analysis.


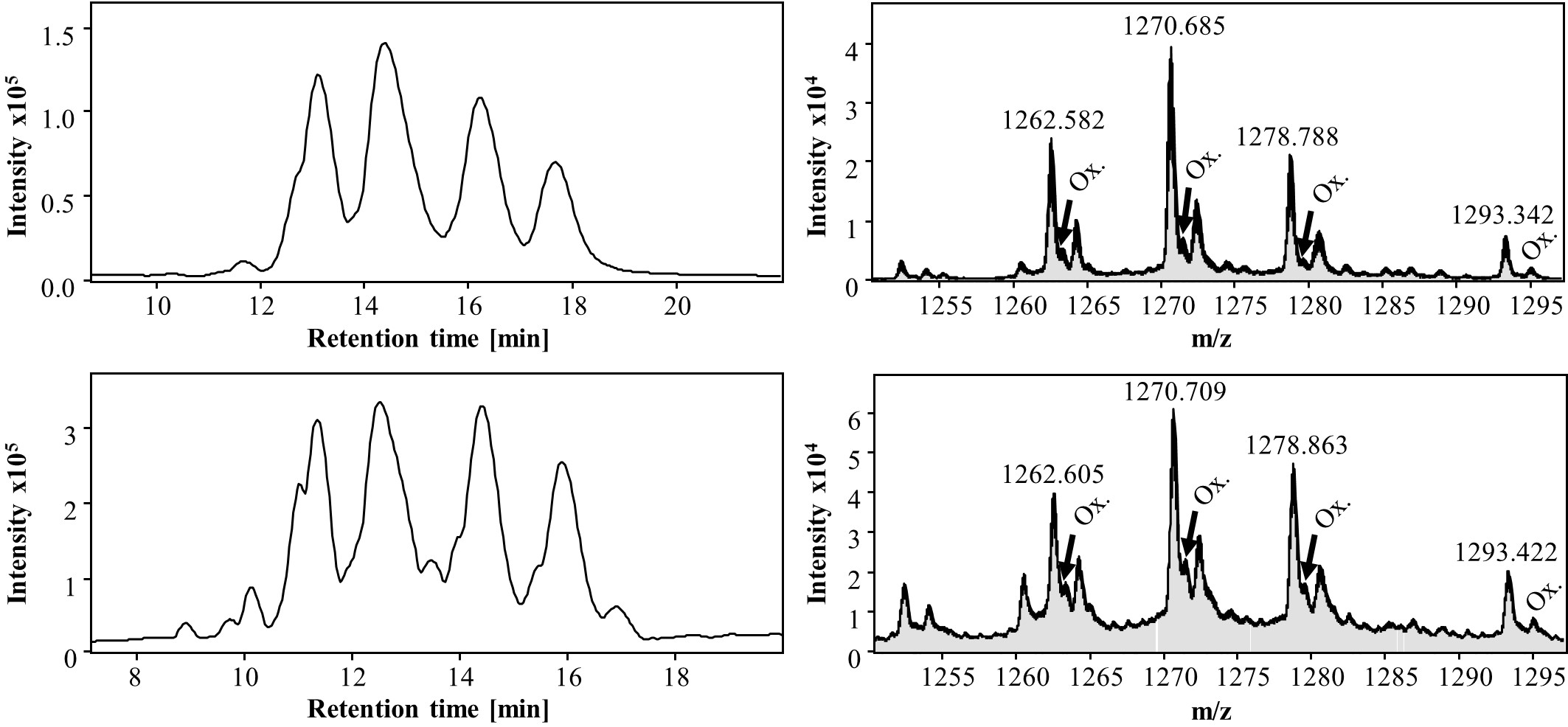


**B**

**D**

**C**

**A**

**Figure S7.** Comparison of BPCs **(A and C)** and corresponding mass spectra of charge state +20 **(B)** from 11-19 min and **(D)** from 10-18 min, obtained during the analysis of Donor 3 before and after a storage period of 9 months. **(A)** Month 0; **(C)** Month 9. After IdeS digestion, Fc/2 subunits were stored dried at -20°C. HILIC-MS conditions: injection of 0.083 mg/mL solution. Temperature 50°C. Linear gradient from 10% to 25% B in 1 min, from 25% to 33% B in 1 min, from 33% to 36% B in 19 min, from 36% to 55% B in 1 min and from 55% to 90% B in 3 min.


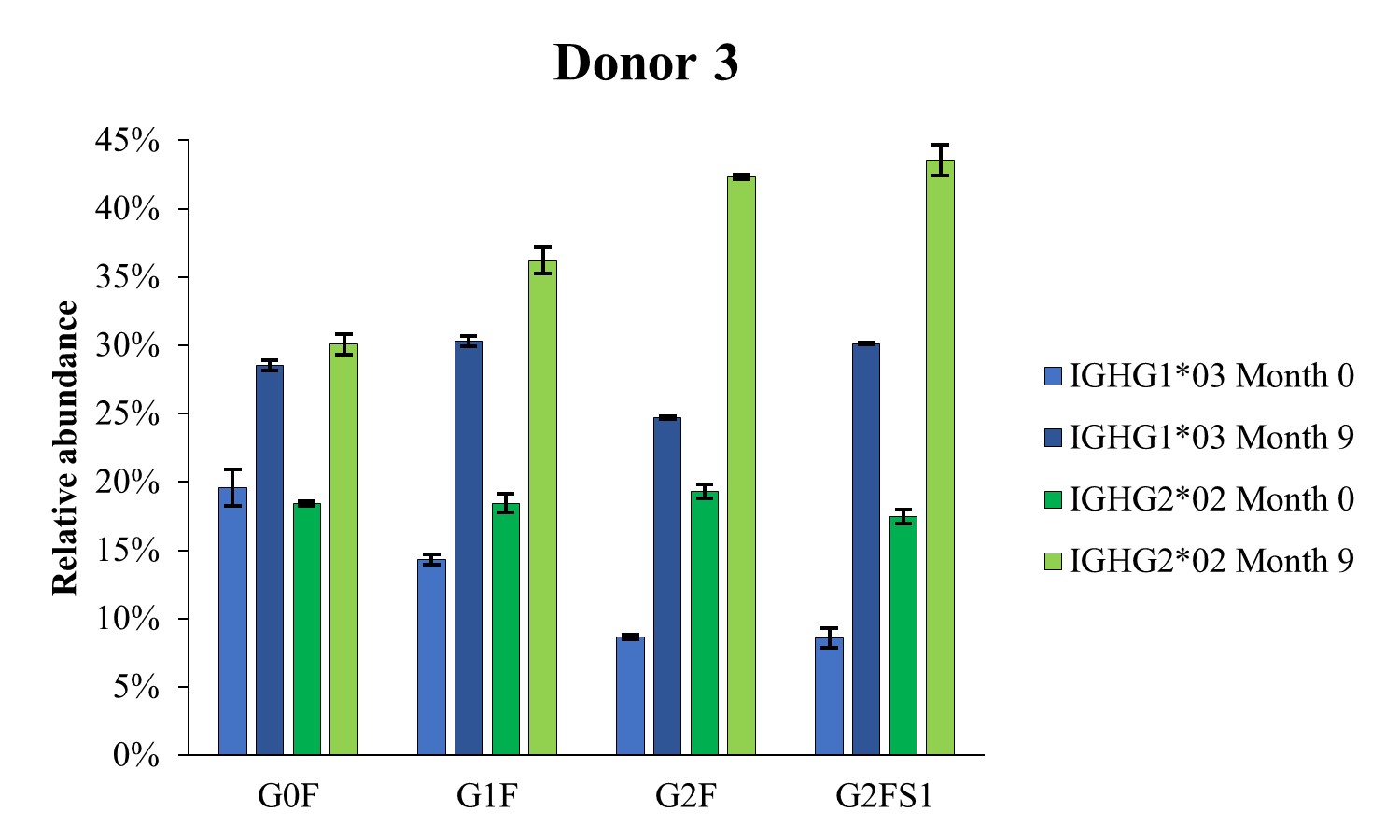


**Figure S8.** Comparison of the oxidation levels for Donor 3 measured before and after a period of 9 months of storage. The relative abundances were calculated using the total area of the four most abundant glycoforms showing the percentage of oxidized glycoform per glycoform. SDs were determined based on the three replicates of each analysis.

**
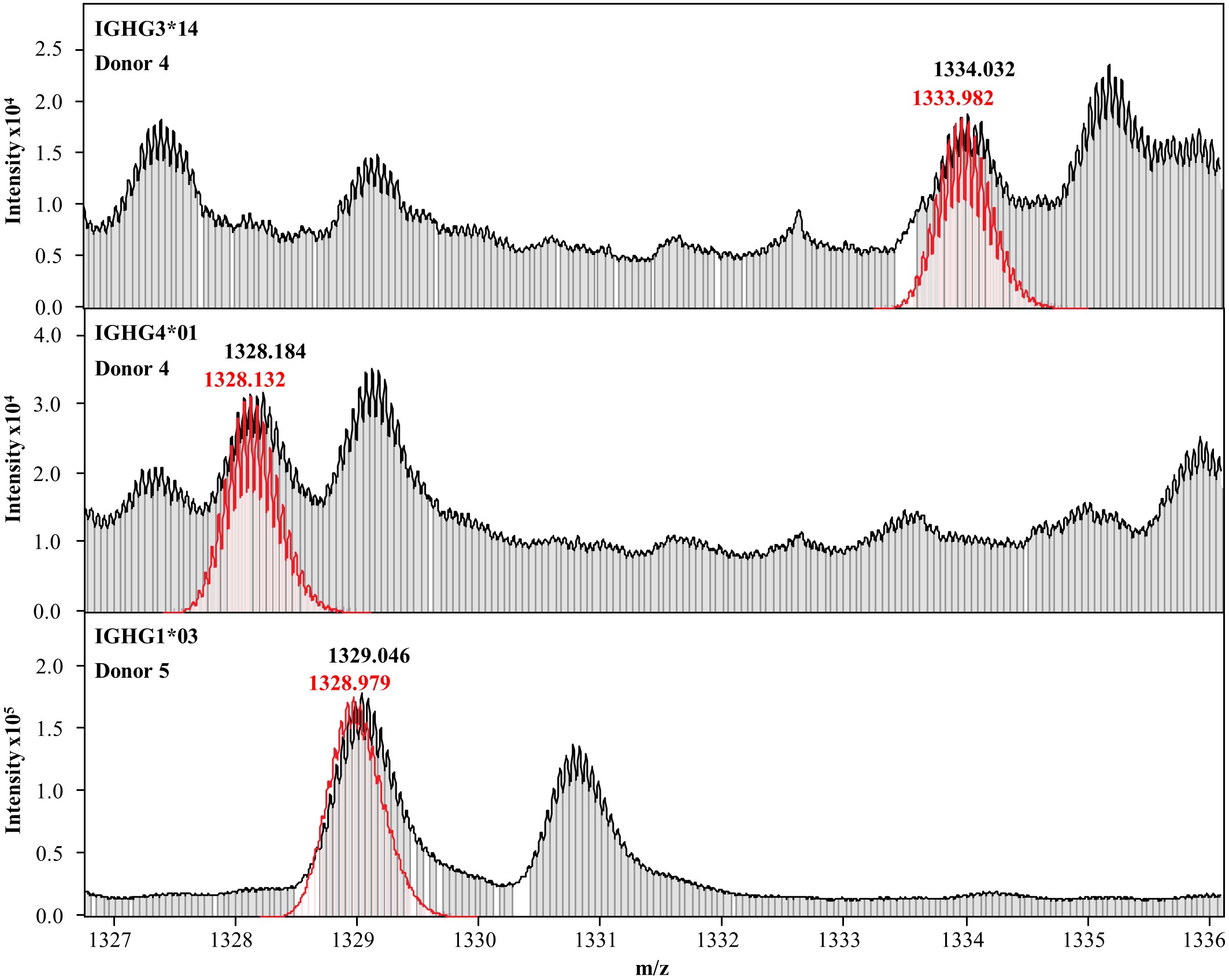
**

**Figure S9.** Comparison between the theoretical isotopic distribution (in red) and the obtained spectra for charge state +19 for the allotypes of Donors 4 and 5 showing a relatively high deviation of theoretical mass (ppm errors showed in Table 1).

**SUPPLEMENTARY TABLES**

**Table S1.** Retention times of the allotypes observed during HILIC-MS for control Donor 5 and its corresponding mAb standards allotypes. The standard deviations were calculated based on the retention times of the three replicates of each analysis of the recombinant allotypes. Each analysis was preceded by the analysis of Donor 5.

| **Allotype** | **Retention time**  **± standard deviation (min)** | **Deviation from control (min)** |
| --- | --- | --- |
| IGHG1*03 (Donor 5) | 12.87 | -0.11 |
| IGHG1*03 | 12.98 ± 0.11 |  |
| IGHG1*07 (Donor 5) | 13.49 | 0.13 |
| IGHG1*07 | 13.36 ± 0.03 |  |
| IGHG2*02 (Donor 5) | 12.59 | 0.03 |
| IGHG2*02 | 12.56 ± 0.08 |  |
| IGHG3*11 (Donor 5) | 11.49 | -0.08 |
| IGHG3*11 | 11.57 ± 0.07 |  |
| IGHG4*01 (Donor 5) | 12.04 | -0.14 |
| IGHG4*01 | 12.18 ± 0.01 |  |

**Table S2.** Relative abundances based on the four main glycoforms and their oxidized variants of all the different allotypes observed for each donor. (*The relative abundances for Donor 2 were calculated without including the allotypes of IgG3 of which the four main glycoforms were not sufficiently resolved). Each analysis was performed in triplicate and the standard deviations calculated.

| **Donor** | **Allotype** | **Relative abundance ± standard deviation (%)** |
| --- | --- | --- |
| 1 | IGHG1*03 | 55.95 ± 0.25 |
|  | IGHG2*02 | 31.71 ± 0.37 |
|  | IGHG3*11 | 3.28 ± 0.05 |
|  | IGHG4*01 | 4.76 ± 0.07 |
|  | IGHG4*02 | 4.30 ± 0.06 |
| 2* | IGHG1*01 | 36.38 ± 0.46 |
|  | IGHG1*03 | 30.22 ± 0.12 |
|  | IGHG2*01 | 11.63 ± 0.02 |
|  | IGHG2*06 | 17.91 ± 0.45 |
|  | IGHG3*01 | N/D |
|  | IGHG3*11 | N/D |
|  | IGHG4*01 | 3.86 ± 0.12 |
| 3 | IGHG1*03 | 63.16 ± 0.44 |
|  | IGHG2*02 | 26.89 ± 0.22 |
|  | IGHG3*11 | 4.30 ± 0.14 |
|  | IGHG4*01 | 2.74 ± 0.14 |
|  | IGHG4*02 | 2.91 ± 0.10 |
| 4 | IGHG1*01 | 34.12 ± 0.11 |
|  | IGHG1*07 | 36.37 ± 0.36 |
|  | IGHG2*01 | 23.01 ± 0.25 |
|  | IGHG3*14 | 2.51 ± 0.08 |
|  | IGHG4*01 | 2.32 ± 0.09 |
|  | IGHG4*03 | 1.68 ± 0.11 |
| 5 | IGHG1*03 | 29.90 ± 0.30 |
|  | IGHG1*07 | 24.24 ± 0.31 |
|  | IGHG2*02 | 34.32 ± 0.15 |
|  | IGHG3*11 | 4.41 ± 0.10 |
|  | IGHG4*01 | 7.13 ± 0.02 |
